# 

**Published:** 1923-01

**Authors:** 


### DIRECTORY

FOR

### Members of the Dental Profession whose practices are limited to a specialty.

The object of this directory is to furnish ready reference to specialists who take referred patients. If you wish your name inserted in this list please correspond with the Dental Register, Box 838, Cincinnati, Ohio.

Your name, specialty, office hours and phone number will be published.

This entire page is reserved for Professional Directory.

### Cincinnati

Dental X-Ray Diagnosis
**CHARLES K. FIELD**
61 Groton Bldg., 7th and Race Sts.
Hours, 10 to 4
Canal 2127

Periodontoclasia (Pyorrhea and Oral Prophylaxis)
**EDGAR H. EBERLE**
605-06 Union Central Bldg., 4th and Vine Sts.
Hours, 9 to 5—Consultation hours by appointment
Main 882

Periodontoclasia (Pyorrhea and Oral Prophylaxis)
**MARIE L. BECKERT**
25 Groton Bldg., 7th and Race Sts.
Hours, 9 to 5—Consultation hours by appointment
Canal 842

Exodontia (Extraction and Oral Surgery)
**ROBERT M. SCHELL**
20 W. 9th St., between Vine and Race Sts.
Hours, 1 to 5—Consultation by appointment
Canal 118

### ANNOUNCEMENTS

### ALUMNI SOCIETY OF THE DEWEY SCHOOL OF ORTHODONTIA

The next annual meeting of this society will be held on April 12th-13th at the Edgewater Beach Hotel in Chicago. The usual high standard of the meetings of this society will be maintained. All interested in Orthodontia are cordially invited to attend these meetings.—George F. Burke, Secretary, 741-43 David Whitney Bldg., Detroit, Michigan.

### ANNOUNCEMENT TO THE DENTAL SUPPLY TRADE

Claudius Ash, Sons & Company (of Canada) Limited and The Temple-Pattison Company, Limited, have joined in a new company to be known as The Ash-Temple Company, Limited, under the management of Mr. H. P. Temple as president; Mr. J. W. Brent, vice president; Mr. W. M. Temple as secretary-treasurer, and Mr. T. H. Temple, director.

The two component companies have in the past invariably provided at all their depots full representation of American goods in demand by Canadian dentists. The Ash-Temple Company, Limited, will continue this policy unchanged. The continued co-operation of American manufacturers of high grade dental supplies is most cordially welcomed.

In January, 1923, The Ash-Temple Company, Limited, will open a branch depot at Ottawa, and in March a branch depot at Montreal, giving the company ten complete dental depots for supply and maintenance service throughout the whole of the Dominion of Canada. This organization provides a unique and unapproached distributive means, enjoying the highest prestige and confidence among the Canadian profession, for offering to Canadian dentists all new and standard products as manufactured and developed by the research and skill of manufacturers and dentists in the advancement of dental science.

Canadian patrons will be pleased to know that the above company carries full line of dental specialties manufactured by The Samuel A. Crocker Company.

---

Drs. Neil and Bowen of Nashville, Tenn., prosthodontists, are giving classes in Bowen-Neil technic. Dr. Neil says that this technic was first given before the Tennessee Association and has since been given before twenty-five state and distinct societies. He has taught classes in five states and has letters from dentists and patients testifying in highest praise of the success of Bowen-Neil technic in fitting of dentures for mouths with flat ridges. Every phase of full denture work is covered and dentists who take this course should be able to return to his office and get results with all those difficult cases.

The Bowen-Neil technic is a mechanical retention of lower dentures secured by extending the lingual flange between the mylo-hyoid muscle and pterygo-mandibular ligament. This technic combined with a modified muscle trim technic gives us the most perfect results possible.

This area lies behind and below the internal oblique line of the retro-molar triangle. It is not a part of the alveolar process and therefore is not involved in the absorptive process following extraction.

The low flat lower ridge (so called complete absorption) is our most favorable case and a denture constructed after this technic for such a case cannot be dislodged by the stress of mastication.

More than three hundred practical cases have been made, all of which have been checked up and are being worn in comfort.

Drs. Neil and Bowen will give instruction to classes in this technic if arrangements can be made through local or district dental societies. Embalmed specimens, lantern slides, models and finished dentures are a part of the complete teaching equipment. For further information address Elsie May Krumpler, Secretary, 226 Lambuth Building, Nashville, Tenn.

